# Associations of Ultrasound Findings with Serum Iron and Ferritin Levels in Children with Obesity

**DOI:** 10.3390/life14040484

**Published:** 2024-04-07

**Authors:** Mioara Desdemona Stepan, Ștefănița Bianca Vintilescu, Claudiu Marinel Ionele, Gheorghe Gindrovel Dumitra, Mihaela Andreea Podeanu, Camelia Cristiana Bigea, Victor Mihai Sacerdoțianu, Cătălina Mihaela Anastasescu, Dan Nicolae Florescu

**Affiliations:** 1 Department of Infant Care-Pediatrics-Neonatology, University of Medicine and Pharmacy of Craiova, 200349 Craiova, Romania; desdemona.stepan@umfcv.ro; 2 Research Center of Gastroenterology and Hepatology, University of Medicine and Pharmacy Craiova, 200349 Craiova, Romania; ioneleclaudiu@gmail.com (C.M.I.); sacerdotianumihai@gmail.com (V.M.S.); dan.florescu@umfcv.ro (D.N.F.); 3 Department of Family Medicine, University of Medicine and Pharmacy of Craiova, 2-4 Petru Rareș Street, 200349 Craiova, Romania; gindrovel.dumitra@umfcv.ro; 4 Doctoral School, University of Medicine and Pharmacy of Craiova, 200349 Craiova, Romania; camelia_bigea@yahoo.com; 5 Hospital of Neuropsychiatry Craiova, Children Mental Health Center, Pharmacology Department, University of Medicine and Pharmacy of Craiova, 200349 Craiova, Romania; catalina_tocea@yahoo.com

**Keywords:** obesity, children, iron, ferritin, ultrasound, echographic, elastographic

## Abstract

The nutritional status of children is always a concern for medical professionals. Increasingly, obesogenic factors have created a new global epidemic. Obesity is characterized by persistent inflammation, which can have detrimental effects on the growth and development of children. Iron and ferritin are both important factors to consider when evaluating these children due to their role in the pathophysiology of chronic inflammation. Recently, ultrasonographic evaluation seems to be an increasingly used method in pediatric clinical practice. In this study, we included 98 children aged 6–14 years, 72 of whom were obese and 26 had normal weight. The data obtained indicated that there was an association between low iron and increased ferritin levels, aspects of non-alcoholic fatty liver visualized by ultrasonography, and the degree of fibrosis assessed elastographically. Ultrasonography can help us identify liver damage, but the possibility of identifying an association with other paraclinical investigations such as iron and ferritin levels can be cumulative. In this way, the assessment can be more complex, as the child benefits from comprehensive evaluation and management. The results drew attention to non-invasive, easy-to-perform, and low-cost methods of assessing obese children in terms of iron metabolism and structural liver changes.

## 1. Introduction

In recent years, there has been a dramatic increase in obesity among people of all ages. The COVID-19 pandemic seems to have exacerbated this through the restrictions imposed but also by the added psychological stress [1]. Recent studies are drawing increasing attention to the risks to which the obese pediatric population is exposed, some of them highly serious with major long-term costs [1,2,3,4].

The treatment of childhood obesity is primarily based on intervention through nutritional therapy and lifestyle changes. These aspects are very often compromised by age. Awareness of the gravity of the situation and the risks they are exposed to from childhood is extremely important. Abdominal functional disorders as well as disorders of the gut microbiome are often associated with obesity and are very expensive to investigate. In this sense, simple, non-invasive, and low-cost methods must be identified in order to be able to observe the aggravating aspects occurring in the body in the context of obesity as early as possible [1,2,5].

The most widely used method in many studies was the use of ultrasound to detect hepatosteatosis. Ultrasound elastography has also been used in various studies to identify fibrosis in the fatty liver in the pediatric population, but due to the lack of clear indications and protocol, it is not broadly used [6,7,8].

Additionally, we sought to identify simple parameters with adjustable values that could serve as a basis for drawing conclusions, leading us to also examine serum iron and ferritin levels. Hepcidin is a peptide hormone involved in iron homeostasis through a regulatory mechanism. Hepcidin is mainly synthesized in the liver but also in other organs including adipose tissue. In this way, the resulting hepcidin links to ferroportin, leading to blockage of cellular iron transport. This explains the increased level of hepcidin identified in obesity. Another mechanism underlying low iron levels in obesity is chronic inflammation; adipose tissue secretes multiple pro-inflammatory cytokines including tumor necrosis factor (TNF)-α and interleukin-6 (IL-6). These contribute to the synthesis of liver proteins including hepcidin [9,10].

Serum ferritin is a marker of iron stores in the body, but it is strongly influenced by inflammatory conditions. There are multiple studies supporting the association between increased ferritin levels and metabolic syndrome and non-alcoholic fatty liver disease (NAFLD) [8,11]. However, we consider it necessary to evaluate and identify ferritin levels in the context of obesity preceding the diagnosis of metabolic syndrome or NAFLD.

The pathophysiological mechanisms of obesity predispose children to chronic inflammation [9,10,11,12] with decreasing serum iron and increasing serum ferritin [11].

Investigation of iron levels together with ferritin levels may help in identifying values considered to be characteristic of obesity, such as low iron and high ferritin levels. Together with these values, ultrasound evaluation can help in deciding the treatment and management of obese pediatric patients. Iron sequestration at the cellular level as well as increased ferritin as an inflammatory marker are important data on the patient’s health status. Multidisciplinary evaluation of pediatric obesity is essential. Routine paraclinical investigations may show liver damage such as hepatosteatosis or even fibrosis, which can be observed by imaging [6,7,10,11].

Our study aimed to identify, using simple and low-cost methods, such as laboratory findings and imaging techniques, the parameters that may indicate structural changes in the liver of children with obesity. More specifically, we tried to identify if there are associations between ultrasonographically assessed liver damage and serum iron and ferritin levels.

## 2. Materials and Methods

### 2.1. Study Design

We conducted a retrospective observational study in order to investigate the relationship between hepatic structural changes and serum iron and ferritin in children with obesity, by analyzing patients’ records. Firstly, we identified all children with obesity and normal weight admitted to the hospital. The next step was to screen them using the inclusion and exclusion criteria stated below. Data collected from patients’ records were age, gender, environment, paraclinical test findings such as ultrasound and elastography findings, as well as serum iron and ferritin levels. Serum iron and ferritin levels were assessed from a “fasting” blood sample, by a routine (automated) analysis. All the included children had ultrasonography and liver elastography results attached to their records.

### 2.2. Study Population

**Figure 1:**
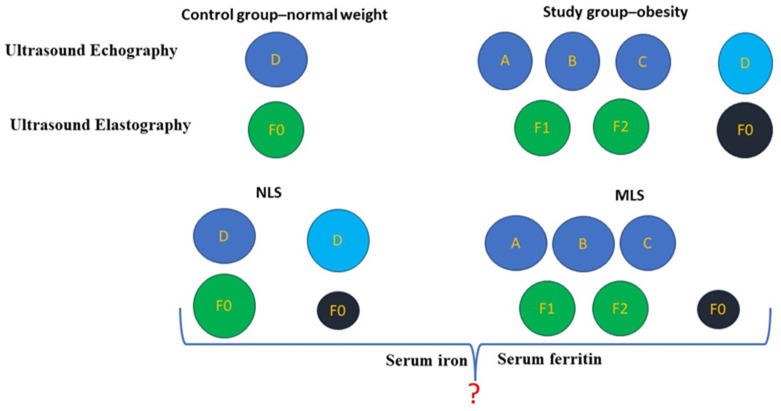
The method of distribution of children according to the assessments performed. (**Score A**) Diffuse increased liver echogenicity, periportal and diaphragmatic echogenicity is still appreciable. (**Score B**) Diffuse increased liver echogenicity hiding periportal echogenicity but still appreciable diaphragmatic echogenicity. (**Score C**) Diffuse increased liver echogenicity hiding periportal and diaphragmatic echogenicity. (**Score D**) Normal liver echogenicity. Grade F0 0–6 kPa Grade F1 6–7 kPa Grade F2 7–8 kPa. There were patients classified as obese group F0 (without hepatic fibrosis) but who showed MSL hepatic stuctural changes from an ultrasound point of view.

We included 98 children aged between 6 and 14 years, 72 (study group) with obesity and 26 (control group) with normal weight. Children were selected and evaluated between September 2021 and April 2022 in the Pediatric Department of Craiova County Emergency Clinical Hospital. After evaluation, children were classified into two groups, the group without hepatosteatosis changes identified by echography, the Normal Liver Structure (NLS) group, and the Modified Liver Structure (MLS) group composed of children with fatty liver-specific changes identified ultrasonographically (Figure 1).

### 2.3. Inclusion Criteria

The inclusion criterion for this study was the presence of obesity (BMI ≥ 95th percentile, in accordance with WHO criteria for gender and age) for the study group, and for the control group, the inclusion criterion was normal weight (BMI ≤ 5th percentile but ≤85th percentile in accordance with WHO criteria for gender and age). An age between 6 and 14 years was an inclusion criterion for both study groups.

### 2.4. Exclusion Criteria

Exclusion criteria were applied for all children represented by the presence of autoimmune or inflammatory diseases, endocrine or hereditary conditions, and other chronic conditions or liver disease. Children with malformations, infectious pathology in the last 3 months, immunocompromised children, or those who had been treated with antibiotics or iron supplements in the last 3 months were also excluded.

### 2.5. Children Assessment

The identification of the non-eligible cases was based on the history of the child. Epidemiological data referring to age, gender, and environment were recorded. Investigation of children included in the study was based primarily on the determination of obesity, i.e., BMI (height, weight, age, gender), liver ultrasound imaging with normal structure changes and serum iron and ferritin levels. In this context, the group with normal weight or without ultrasonographically visible liver structural changes constituted the control group.

### 2.6. Measurements

An anthropometric investigation was performed using standardized measurement methods. Measurements were taken in the morning after awakening. For height, a thalliometer with 1 mm graduation was used, and for weight measurement, we used an electronic scale (100 g graduation). Body mass index was calculated by dividing body weight (expressed in kilograms) by height squared (expressed in meters), taking into account gender and age.

### 2.7. Laboratory Tests

A fasting venous blood sample (6 mL) was collected from each participant and analyzed for serum iron and serum ferritin. Iron deficiency was considered below reference values as follows: <14 years: 29–137 μg/dL; boys: 14–19 years: 43–176 μg/dL; girls: 14–19 years 33–170 μg/dL; serum iron was measured by a colorimetric method. The determination method for serum ferritin was immunochemical with electrochemiluminescence detection (ECLIA) with the following reference values: 4–7 years: 4–67 ng/mL; 7–13 years: male 14–124 ng/mL, female 7–84 ng/mL; 13–18 years: male 14–152 ng/mL, female 13–68 ng/mL 7–142 ng/mL. Both serum iron and ferritin indirectly evaluate the inflammation existing in obesity.

### 2.8. Imagistic Result

Imaging data were obtained using ultrasound echography with age- and fat-adapted scanning transducers. In order to quantify liver fat deposition, we classified children as follows: score A, diffuse increased liver echogenicity, periportal and diaphragmatic echogenicity are still appreciable; score B, diffuse increased liver echogenicity hiding periportal echogenicity but diaphragmatic echogenicity is still appreciable; score C, diffuse increased liver echogenicity hiding periportal and diaphragmatic echogenicity and score D normal liver echogenicity. For the quantification of liver fibrosis, we performed elastography for which an adapted sensor was also used, and by determining 10 valid measurements, a mean value expressed in kiloPascals was indicated. This allowed us to quantify the degree of fibrosis as follows: 0–6 kPa—grade F0; 6–7 kPa—grade F1; 7–8 kPa—grade F2.

### 2.9. Statistical Analysis

Analysis of the clinical–epidemiological data was performed using the chi-square comparison test (χ^2^) from SPSS10 (Statistical Package for Social Sciences), where a *p*-value < 0.05 was considered significant. This study also made assessments about the tendency of association between different parameters, even if the relationships were without significant *p*-values.

### 2.10. Ethical Principles

Our study respected the guidelines of the Declaration of Helsinki. Written informed consent was obtained from legal representatives of all the children when they were included in the study from the grant “Utility of antioxidants in the prevention, monitoring, and treatment of non-alcoholic fatty liver disease in children with established PNPLA3 genotype”, from which all of the children included in this study were selected. Approval of the Local Ethics Committee with registration No. 118/19.07.2021 was obtained.

## 3. Results

The clinico-epidemiological analysis of the study group included 98 children; 72 cases (73.5%) of the children were classified as obese, and 26 cases (26.5%) had normal weight for age and gender, representing the control group. In total, 48 cases (49%) fell into the age category of 6–9 years, and 50 cases (51%) were aged 10–14 years. The majority were males with urban provenance, with 58 cases (59.2%) and 51 cases (52%), respectively.

The classification of the children according to the parameters analyzed revealed that obesity with BMI 27 ± 3.8 was more common in the age category 10–14 years, male and urban origin, compared to the control group, where BMI was 16.7 ± 1.2 and children in the age category 6–9 years and rural origin dominated. These aspects were not statistically associated (Table 1).

Imaging investigation revealed that the control group with normal body weight did not show any ultrasonographically detectable changes in the liver. All children had fibrosis grade F0 with mean 2.9 ± 0.5 kPa, included in the D score, without hepatic steatosis changes. In the group of children with obesity, the mean increased to 5.1 ± 1.7 kPa; most of them had fibrosis grade F0, with statistical association in relation to obesity (Table 1).

In relation to the imaging investigation, the degrees of hepatosteatosis identified demonstrate the heterogeneity of the group, including children with obesity but without changes in hepatosteatosis (22 cases, 30.6%). From this point of view, the statistical analysis also demonstrated the association between obesity and imaging results visualized in terms of hepatic steatosis (Table 1).

The paraclinical investigation of iron and ferritin levels showed a dramatic decrease in iron and a clear increase in ferritin in obese children compared to the control group, which were also statistically associated. There were no cases with increased sideremia or cases with decreased ferritin (Table 1).

Redistributing the children according to the structural changes identified in the liver, we obtained the groups MLS for 50 cases (51.1%) and NLS for 48 cases (48.9%). We observed that all the children in the control group with normal weight fitted into the NLS group, but 22 cases of obese children representing 22.4% also fitted into the NLS group.

Liver structural changes were not associated with the age of the children but were statistically associated with obesity (MLS-BMI 27.7 ± 4.2 compared to NLS-BMI 20.7 ± 4.7) and with iron deficiency and hyperferritinemia. A percentage of 48.9 (50 cases) with iron deficiency and hyperferritinemia was identified in the MLS children. Serum iron levels were much lower in the MLS group compared to the NLS group, at 10.5 ± 5.2 μg/dL and 45.4 ± 27.6 μg/dL, respectively. In the same way, an increase in serum ferritin was observed in MLS and NLS, with 201.7 ± 28 ng/mL and 97.6 ± 60.8 ng/mL, respectively (Table 2).

**Figure 2:**
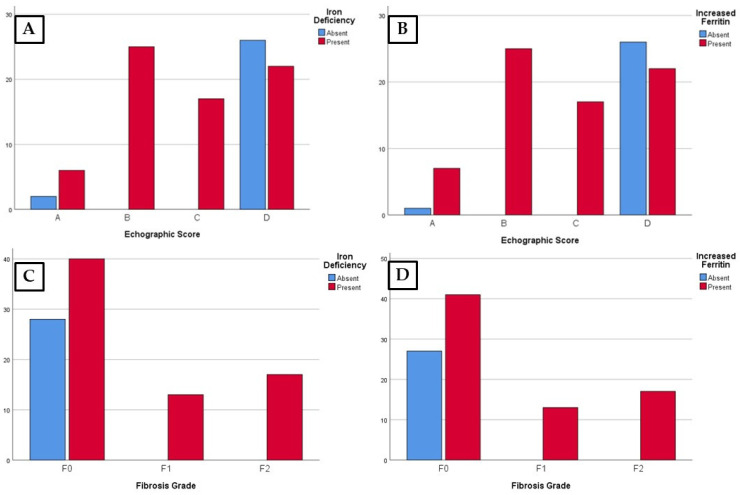
The distribution of cases according to iron deficiency and imaging evaluation (**A**,**C**) and increased ferritin and imaging evaluation (**B**,**D**).

In the ultrasound imaging investigation, we found that low iron levels were predominantly identified in children in the MLS group. Score A was found in 6.1% of the cases, score B was found in 25.5% of cases, and score C was found in 17.3% of cases. Increased ferritin levels were also identified in children classified by ultrasound scores with structural changes of hepatosteatosis. The data showed statistical associations between liver structural changes identified echographically and low iron and increased ferritin levels, respectively (Table 3, Figure 2A–D).

In terms of serum iron and ferritin levels and liver structural changes identified by elastographic examination, statistical associations were found between iron deficiency and increased serum ferritin values and the degree of elastographic changes. It was observed that children with liver structural changes classified as F1 and F2 fibrosis grades were identified only in the iron deficiency or increased ferritin level groups (Table 4, Figure 2).

Iron deficiency and ferritin elevation were statistically associated with both echographic and elastographic imaging changes.

## 4. Discussion

Obesity is an increasingly used term in the medical world. Its escalation worldwide has even turned it into an epidemic notion. Its recognition at younger and younger ages highlights its repercussions on the body. Obese children and adolescents are potential candidates for serious adult diseases such as cardiovascular disease or even cancer [8].

Recent studies in the area have focused more on research into non-alcoholic fatty liver disease (NAFLD), which is the first stage of fat accumulation in the liver. Investigation of NAFLD has brought numerous paraclinical methods of evaluation to the attention of medical practice, but it requires a rather long follow-up time [13]. Early assessment of the progression from NAFLD to steatohepatitis (NASH), cirrhosis, and even hepatocellular carcinoma is extremely important, which is why we consider early evaluation of children with common and accessible methods to be a necessity [8,14,15].

The global prevalence of obesity has increased significantly in the 5–19 age group, by about eight times, in both females and males, according to a study by Lister et al. published in May 2023 [16]. This is a worrying fact, especially as the same study highlights variations in obesity prevalence depending on factors influencing the obesogenic environment, especially economic factors. From the data found in the literature, it appears that the male sex seems to be more affected by obesity without being clear why, which is a fact also identified in our study. We noticed that metabolic syndrome, which is the foundation of obesity, is also more present in males according to a Systematic Literature Review published in 2023 [16,17,18]. Concerning the environment of origin, the urban environment favors obesogenic factors by the increased availability of low-cost foods with increased kcal and convenience [16].

Echography, as a large-scale ultrasonographic method, helped us to clearly identify the changes in hepatosteatosis that were statistically associated with obesity, which was expected. With the help of this study, we observed that the control group but also one-third of the study group had no changes in this area. However, there was a considerably increased difference between the percentages of children with hepatosteatosis and those with liver fibrosis. This, on the one hand, proved the heterogeneity of the group, and on the other hand, it pointed to the idea of an evolutionary cascade of fat accumulation in the liver. There is a multitude of studies that have demonstrated the usefulness of ultrasound as a common screening method for the evaluation and monitoring of pediatric patients with NAFLD, but it is considered inaccurate in the absence of experience [19,20,21].

An article published in 2023 by Močnik M. et al., *Ultrasound Elastography in Children*, clearly describes the usefulness of elastography in the evaluation of children [6]. Elastography is a new, non-invasive, feasible technique for quantifying fibrotic changes in the liver of children with obesity [7,22]. Our study shows that in the control group, we did not identify liver fibrosis. In the group of obese children, most of them had no fibrotic changes in the liver, but the percentages identified in the F1 and F2 stages should not be ignored, taking into account the age of the patients. Regarding age, we consider this fact to be of major importance, although the percentages were quite low. The statistical association proved to be significant, and it was observed that obesity clearly predisposes to fibrotic changes in the liver [6,7,22].

A systematic review published in 2023 by Pedro Ferro Berton et al. focuses attention on the chronic inflammation induced by obesity, which leads to increased hepcidin levels through IL-6 production [9]. Also, Alshwaiyat N.M. et al. explain details related to low iron levels in obesity [10]. Low iron levels in obesity are, on the one hand, due to iron sequestration in macrophages and, on the other hand, to decreased absorption resulting from increased hepcidin. Furthermore, it is underlined that obese children should not receive iron even though they have low levels. According to pathophysiological mechanisms, weight loss improves serum iron levels as well [9,10,12,23], which is also highlighted by our research.

In our study, we observed the same: serum iron levels were decreased in obese children with a mean of 14.5 ± 11.9 μg/dL compared to the control group 63.8 ± 20.5 μg/dL, with a statistical association.

Recently, serum ferritin levels have been studied in an attempt to demonstrate the predictability of hepatic structural changes based on increasing ferritin levels. A meta-analysis performed in 2023 by Yan J et al. highlights this point [24]. The information that serum ferritin levels are directly proportional to hepatic fat storage and associated inflammation, independent of iron stores in the body, has been confirmed by other research [8,24,25]. The data from our study are aligned with the information found in the literature [24]: serum ferritin levels were considerably higher in obese children compared to normal-weight children: 187.2 ± 36.2 ng/mL and 49.5 ± 29.5 ng/mL respectively.

Based on these aspects identified in other research, specifically ultrasonography, either echographic or elastographic, used as a method of evaluation of obese children and quantification of serum iron and ferritin levels, we tried to observe other statistical associations. In this direction, we noticed that hepatic structural changes (MLS) are not identified in all obese children, highlighting the fact that not all obese pediatric patients have hepatosteatosis, much less fibrosis. Following the same protocol, we identified low iron levels and increased ferritin levels in MLS children compared to those with Normal Liver Structure (NLS).

Iron deposition in obese patients occurs in both hepatocytes and macrophages and is often referred to as dysmetabolic iron overload syndrome. Iron is involved in the catalytic reaction and formation of toxic hydroxyl radicals that can increase the progression of fibrosis in the liver. Research is more concerned with the study of pediatric patients with NAFLD, but obesity is a precondition that deserves to be investigated more thoroughly [8,24,26,27].

In the majority of studies quantifying hepatosteatosis, methods such as MRI or even liver biopsies have been used, which are costly or invasive methods [8,24,27]. Data obtained in this study demonstrated statistical relations between liver structural changes identified by both echographic and elastographic methods, low serum levels of iron, and increased serum levels of ferritin.

It is important to note that the children classified with F1 and F2 fibrosis are children who only have iron deficiency or hyperferritinemia. The identified aspects contribute to maintaining the hypothesis that changes in iron and ferritin homeostasis definitely appear at the time of fibrosis. According to the pathophysiological mechanisms, the onset of serum changes already occurs in the phase of obesity without hepatic structural changes. This idea is confirmed by the data provided in this study; thus, both echographic and elastographic statistical associations were identified [28,29,30,31].

Our study has some limitations. The study design was cross-sectional and, therefore, we could not identify a clear causal relationship between serum markers and liver structural changes. Also, we could not obtain social data that may influence the incidence of liver structural changes. Last but not least, the liver structural changes were not confirmed histologically or by MRI. In order to support our point of view, we considered it necessary to look at the problem through two ultrasonographic methods. At the same time, the heterogeneity of the group can be a plus point.

## 5. Conclusions

At a routine examination in the doctor’s consulting room, obese pediatric patients with low iron levels identified in routine tests should receive more attention. Low serum iron levels and increased serum ferritin levels may be markers in the evaluation of obese children in association with echographic and elastographic evaluation. These levels do not necessarily mean iron deficiency anemia and may raise the suspicion of progressive hepatosteatosis. Thus, in search of easy methods to identify potential candidates for NAFLD or NASH, we can try serum ferritin determination paired with non-invasive imaging methods in evaluation and monitoring. These methods are limited mainly by the experience of the examiner, but, considering their cost, time, and non-invasive nature, they may represent an alternative investigation.

## Figures and Tables

**Table 1:** The distribution of cases in relation to the analyzed parameters.

Parameters% (No. Cases) (%)			Normal Weight	Obesity	*p* Value (x^2^ Test)
Age	6–9 years		14 (53.8%)	34 (47.2%)	*p* > 0.05
	10–14 years		12 (46.2%)	38 (52.8%)	
Gender	Female		11 (42.3%)	29 (40.3%)	*p* > 0.05
	Male		15 (57.7%)	43 (59.7%)	
Environment	Urban		10 (38.5%)	41 (56.9%)	*p* > 0.05
	Rural		16 (61.5%)	31 (43.1%)	
BMI			16.7 ± 1.2	27 ± 3.8	
Ultrasound Elastography	kPa		2.9 ± 0.5	5.1 ± 1.7	*p* < 0.001
	Fibrosis score	F0	26 (100%)	42 (58.3%)	
		F1	0	13 (18.1%)	
		F2	0	17 (23.6%)	
Ultrasound Echography	Score A		0	8 (11.1%)	*p* < 0.001
	Score B		0	25 (34.7%)	
	Score C		0	17 (23.6%)	
	Score D		26 (100%)	22 (30.6%)	
Serum iron	μg/dL		63.8 ± 20.5	14.5 ± 11.9	*p* < 0.001
	Deficit		3 (11.5%)	67 (93.1%)	
Serum ferritin	ng/mL		49.5 ± 29.5	187.2 ± 36.2	*p* < 0.001
	Increased		2 (7.7%)	69 (95.8%)	

BMI: body mass index; kPa: kiloPascal.

**Table 2:** The distribution of cases in relation to liver structural changes and the analyzed parameters.

Parameters% (No. Cases) (%)		NLS	MLS	*p* Value
Age (years) 6–9 years 10–14 years		9.4 ± 2	9.8 ± 2.2	*p* > 0.05
		23 (47.9%)	25 (50%)	
		25 (52.1%)	25 (50%)	
BMI		20.7 ± 4.7	27.7 ± 4.2	*p* < 0.001
Normal weight		26 (26.5%)	0	
Obesity		22 (22.4%)	50 (51.1%)	
Serum Fe	μg/dL	45.4 ± 27.6	10.5 ± 5.2	*p* < 0.001
	Deficit	22 (22.4%)	48 (48.9%)	
Serum Ferritin	ng/mL	97.6 ± 60.8	201.7 ± 28	*p* < 0.001
	Increased	22 (22.4%)	49 (50%)	

NLS: Normal Liver Structure; MLS: Modified Liver Structure.

**Table 3:** The distribution of cases in relation to the ultrasound assessment and iron and ferritin levels.

Parameters% (No. Cases) (%)			Ultrasound Echography				*p* Value
			MSL			NSL	
		Total	Score A	Score B	Score C	Score D	
Serum iron	Normal level	28 (28.6%)	2 (2.1%)	0	0	26 (26.5%)	*p* < 0.001
	Deficit	70 (71.4%)	6 (6.1%)	25 (25.5%)	17 (17.3%)	22 (22.5%)	
Serum ferritin	Normal level	27 (27.5%)	1 (1%)	0	0	26 (26.5%)	*p* < 0.001
	Increased	71 (72.4%)	7 (7.1%)	25 (25.5%)	17 (17.3%)	22 (22.5%)	

NLS: Normal Liver Structure; MLS: Modified Liver Structure.

**Table 4:** The distribution of cases in relation to the elastographic assessment and iron and ferritin levels.

Parameters/% (No. Cases) (%)			Ultrasound Elastography			*p* Value
		Total	F0	F1	F2	
Serum iron	Normal level	28 (28.6%)	28 (28.6%)	0	0	*p* < 0.001
	Deficit	70 (71.4%)	40 (40.8%)	13 (13.2%)	17 (17.3%)	
Serum ferritin	Normal level	27 (27.5%)	27 (27.5%)	0	0	*p* < 0.001
	Increased	71 (72.4%)	41 (41.8%)	13 (13.2%)	17 (17.3%)	

## Data Availability

The data presented in this study are available on request from the corresponding author.

## Abbreviations

COVID-19	Coronavirus disease 2019
TNF-α	Tumor necrosis factor
IL-6	Interleukin-6
NAFLD	Non-alcoholic fatty liver disease
NLS	Normal Liver Structure
MLS	Modified Liver Structure
BMI	Body mass index
WHO	World Health Organization
NASH	Non-Alcoholic Steatohepatitis
MRI	Magnetic Resonance Imaging
